# Individual differences in resilience to stress are associated with affective flexibility

**DOI:** 10.1007/s00426-022-01779-4

**Published:** 2022-12-17

**Authors:** Lena Rademacher, Dominik Kraft, Cindy Eckart, Christian J. Fiebach

**Affiliations:** 1 Department of Psychology, Goethe University, Frankfurt, Germany; 2 Department of Psychiatry and Psychotherapy, University of Lübeck, Lübeck, Germany; 3 Department of Psychiatry and Psychotherapy, Tübingen Center of Mental Health, University of Tübingen, Tübingen, Germany; 4 Department of Psychiatry, Psychosomatic Medicine and Psychotherapy, University Hospital Frankfurt - Goethe University, Frankfurt, Germany; 5 Brain Imaging Center, Goethe University, Frankfurt, Germany

## Abstract

Cognitive flexibility is frequently linked to resilience because of its important contribution to stress regulation. In this context, particularly affective flexibility, defined as the ability to flexibly attend and disengage from affective information, may play a significant role. In the present study, the relationship of cognitive and affective flexibility and resilience was examined in 100 healthy participants. Resilience was measured with three self-report questionnaires, two defining resilience as a personality trait and one focusing on resilience as an outcome in the sense of stress coping abilities. Cognitive and affective flexibility were assessed in two experimental task switching paradigms with non-affective and affective materials and tasks, respectively. The cognitive flexibility paradigm additionally included measures of cognitive stability and spontaneous switching in ambiguous situations. In the affective flexibility paradigm, we explicitly considered the affective valence of the stimuli. Response time switch costs in the affective flexibility paradigm were significantly correlated to all three measures of resilience. The correlation was not specific for particular valences of the stimuli before or during switching. For cognitive (non-affective) flexibility, a significant correlation of response time switch costs was found with only one resilience measure. A regression analysis including both affective and cognitive switch costs as predictors of resilience indicated that only affective, but not cognitive switch costs, explained unique variance components. Furthermore, the experimental measures of cognitive stability and the rate of spontaneous switching in ambiguous situations did not correlate with resilience scores. These findings suggest that specifically the efficiency of flexibly switching between affective and non-affective information is related to resilience.

**Supplementary Information:**

The online version contains supplementary material available at 10.1007/s00426-022-01779-4.

## Introduction

More than 500 million people world-wide suffer from stress-related mental disorders such as depression or anxiety disorders (World Health Organization 2017). Despite extensive research in the last decades, the high prevalence could so far not be reduced. As one response to this situation, an alternative research strategy has emerged that focuses on improving our understanding of the mechanisms of resilience, i.e., of how some people maintain health in the face of stress and adversity (e.g., Bonanno 2004; Kalisch et al. 2017; Southwick et al. 2005). Resilience has variably been defined as a fixed individual predisposition which is juxtaposed to vulnerability, as the process of adapting well in the face of significant stressors or traumatic experiences, or as the ability to overcome stressful events, i.e., as a healthy outcome after the experience of stress or adversity (see, e.g., Luthar et al. 2000, and Kalisch et al. 2017, for overviews of different definitions). Due to the complexity of the construct, resilience is difficult to measure, and the operationalization has varied considerably in previous research. For example, some researchers have focused on the adaptation to a single extremely stressful and potentially traumatic event such as a terror attack or war experience (Bonanno et al. 2004), whereas others have focused on the ability to overcome accumulated negative life events or daily stressors (e.g., Almeida 2005).

One psychological factor that has been linked consistently to resilience is cognitive flexibility – the capacity to flexibly adjust behavior to changing situational demands (see, e.g., Haglund et al. 2007; Southwick & Charney 2012; see also Parsons et al. 2016, for a discussion of the possible role of psychological flexibility and other cognitive factors for resilience). Cognitive flexibility is often considered as one among a limited set of fundamental executive control functions that are critical for controlled goal-directed cognition and behavior. For example, Miyake and colleagues (2000), referring back to earlier work from experimental psychology (Monsell 1996), proposed that “shifting back and forth between multiple tasks, operations, or mental sets” (p. 55) is one of three core executive control functions (together with processes of inhibition and working memory). This type of mental set shifting involves the disengagement from a currently relevant task set and the activation or implementation of a new set of task rules. In the context of resilience research, cognitive flexibility has particularly often been associated with the ability to flexibly regulate emotions (e.g., Haglund et al. 2007). It has been argued that the capacity to stop inefficient emotion regulation (ER) and to switch to more efficient ER strategies relies on a flexible mode of cognitive control (see Pruessner et al. (2020) for a cognitive control framework of ER flexibility). As flexible emotion regulation is essential for successful adaptation to stress, this framework offers an important link to how flexibility in executive control might contribute to resilience. However, in the context of affective disorders and emotional dysregulation, it has repeatedly been shown, that measures of executive control provide a better predictor of vulnerability and psychopathology when the stimuli being used are affective in nature (Joormann et al. 2010). Accordingly, it remains an open question so far whether mental health (and thus resilience) benefits from cognitive flexibility in general or more specifically from the ability to flexibly handle emotions and / or affective material.

### Cognitive vs. affective flexibility

Cognitive flexibility is most frequently studied with experimental paradigms that require instructed switches between two task rules applied to the same type of stimuli (e.g., Monsell 2003). Switching between tasks requires additional cognitive resources as compared to repeating the same task, which is behaviorally reflected in *task switching costs* — prolonged response times and an increased proneness to commit errors (e.g., Monsell 2003). These switch costs vary between persons, which suggests that individuals differ in how efficiently they can implement and execute the cognitive processes associated with shifting task sets (see Ueltzhöffer et al. 2015, for an exploration of potential neurobiological mechanisms underlying individual differences in task switching efficiency). Interestingly, switch costs correlate with the rate of spontaneous switching in ambiguous situations —which may indicate that switch cost variability is also related to dispositional differences in the proneness to cognitive flexibility (Armbruster et al. 2012). The proposal that cognitive flexibility is associated with resilience to stress (Haglund et al. 2007; Southwick & Charney, 2012) suggests that greater mental flexibility, or a greater efficiency of the mental processes involved in flexibly engaging and disengaging from different task demands, helps to regulate stress and negative emotions more effectively. However, empirical investigations that directly link empirical measures of cognitive flexibility to resilience have so far been scarce.

Inspired by the proposal that cognitive flexibility promotes mental health by enabling effective emotion regulation, recent studies have started to investigate the cognitive mechanisms of flexibly attending to and disengaging from affective information. In analogy to the long-established construct of cognitive flexibility, this has been termed ‘affective flexibility’ (Malooly et al. 2013), but has for example also been introduced as ‘attentional control capacity for emotion’ (Johnson 2009a,b). Experimental tasks assessing affective flexibility usually involve switching between affective and affectively neutral stimuli, stimulus features, and/or tasks, thereby requiring shifts of attention either towards affective information or away from affective information (e.g., Dierolf et al. 2016; Malooly et al. 2013; Reeck & Egner 2015). Affective task switching costs were shown to be inversely associated with the individual’s proneness to rumination (Genet et al. 2013) and to predict the ability to downregulate negative emotions during a sad movie (Malooly et al. 2013). These findings support the proposal that affective flexibility – possibly via its importance for effective emotion regulation – promotes resilience (Genet & Siemer, 2011).

### Cognitive and affective flexibility and their relationship to resilience

The relationship between cognitive and affective flexibility on the one hand and resilience on the other hand has only recently been taken into focus. At the time of preregistration of the research reported here, only one published study had explored the association between cognitive and affective flexibility and trait resilience: Genet & Siemer (2011) examined 64 healthy subjects who filled out two trait resilience self-report scales, i.e., the Connor-Davidson Resilience Scale (CD-RISC, see below) and the Ego-Resiliency Scale (ER89; see Block & Kremen 1996), and who additionally performed two experiments assessing cognitive and affective flexibility. In the cognitive flexibility task, participants switched between two simple numeric tasks (i.e., parity and magnitude judgments on single digits). In the affective paradigm, negative and positive words were presented sequentially, accompanied by a cue indicating either a non-affective task (categorization of words as adjectives vs. nouns) or an affect-related task (categorization of words as having positive vs. negative valence). For both paradigms, switch costs (increased response times for switch compared to repeat trials) were negatively correlated with a combined resilience score calculated from the two questionnaires. Thus, higher trait resilience was associated with more efficient task switching, i.e., higher flexibility. (Note that in the affective paradigm this was only found for ‘inconsistent’ experimental blocks in which the responses of the two tasks were mapped onto different keys in each trial. This, however, may simply reflect that the response button mapping in ‘consistent’ trials did not require the inhibition of incompatible motor responses —because both tasks mapped the correct response on the same response button — and thus could in fact be solved correctly without any reconfiguration of cognitive task sets.) Working memory capacity was not associated with resilience, which the authors interpret as resilience being “tied to specific cognitive processes rather than overall better cognitive functioning” (Genet & Siemer 2011, p. 380). Furthermore, a multiple regression analysis revealed that affective flexibility accounted for variance in trait resilience also when controlling for cognitive flexibility and working memory capacity, suggesting at least partially unique contributions of affective flexibility to trait resilience.

Further studies report results pointing in the same direction as those of Genet & Siemer (2011): Hildebrandt et al. (2016) investigated whether the flexibility to switch between affective and non-affective task sets, heart rate variability, and/or self-reported resilience (ER89) modulate the regulation of arousal in a threating environment. These authors reported a correlation between resilience scores and switch costs when switching to an affective task (evaluation of the valence of a word), but only in case of stimuli with positive valence. Note, however, that switch costs were not calculated in the traditional way in that study. In another study, Grol and De Raedt (2018) showed that persons with higher resilience scores (Dutch version of the Resilience Scale/RS-nl; Portzky et al. 2010) tended to show more efficient task switching when negative information was preceded by positive information. To summarize, while the existence of an association between affective or cognitive flexibility and psychological resilience is highly suggestive, empirical support is so far scarce and the role of important boundary conditions (like the nature of the task or the valence of stimuli) is at present not sufficiently well understood.

### The present study

The present pre-registered study aimed at replicating the work by Genet & Siemer (2011) and extending their results concerning the relationship between psychological flexibility and resilience. For this purpose, three resilience questionnaires were applied which were designed against the background of different definitions of resilience (i.e., as personality trait or outcome, for more details see below). Furthermore, two experimental paradigms were used that assess the efficiency of cue-instructed task switching with emotionally neutral stimuli (cognitive flexibility) and with affective stimuli (affective flexibility): To quantify cognitive flexibility, a paradigm was adapted from Armbruster et al. (2012) which involves switching between a magnitude judgment (greater vs. lower than 5) and a parity judgment (odd vs. even) on visually presented digits. In addition to switch costs, the task also allows to calculate a measure of cognitive stability (reflecting how well a distractor stimulus can be inhibited when participants are instructed not to switch tasks) and the rate of spontaneous switching in ambiguous situations, potentially reflecting individual differences in the proneness to cognitive flexibility (see also Armbruster-Genç et al. 2016; Ueltzhöffer et al. 2015). Affective flexibility was assessed with a variant of this task in which participants were presented with images of happy and angry faces and in which they judged either the valence of the facial expression (affective task) or the gender of the displayed person (neutral task; see Eckart et al. 2021, for psychometric properties of this task). Here, we examined the association between resilience and affective flexibility by considering (i) switch costs separately for both switch directions (i.e., from the affective to the non-affective task and vice versa). We also considered (ii) the valence of the stimuli from which participants are switching away (i.e., of the stimulus preceding the switch) and (iii) the valence to which participants are switching (i.e., of the current stimulus), as factors potentially influencing switch costs.

To investigate the association between resilience and cognitive and affective flexibility, we analyzed behavioral data from the two flexibility tasks (task data available under https://osf.io/8f4cn) and additional questionnaire data obtained from the same persons (available under https://osf.io/v6y39/). Please note that the task data (but not the questionnaire data) are also part of another publication (Kraft et al. 2020) on a different research question that was independently pre-registered (https://osf.io/fuyed). Importantly, both preregistrations were deposited at the Open Science Framework (OSF) prior to the start of data acquisition. Here, we address the following specific research questions and hypotheses (pre-registered under https://osf.io/u47yg): First, are cognitive and affective flexibility related to self-report measures of (trait or outcome-related) resilience? Following Genet & Siemer (2011), we hypothesized that cognitive and affective response time switch costs are negatively correlated with self-reported resilience (Hypothesis 1). As more explorative hypothesis (given lack of prior evidence), we expected that error rate switch costs in both paradigms are also negatively correlated with self-reported resilience (Supplementary Hypothesis 1a). Furthermore, we also hypothesized that this association is specific to flexibility, such that cognitive stability (i.e., distractor inhibition costs) should be either not related to resilience or inversely related (Supplementary Hypothesis 1b). Existing resilience questionnaires are based on slightly different theoretical conceptualizations (for example trait vs. outcome based definitions of resilience). As exploratory analyses, we also examined potential differences in the associations between different resilience questionnaires and experimental markers of flexibility. Second, can a relationship with resilience also be found with spontaneous switching in ambiguous task situations? Given that we have proposed in earlier work that the rate of spontaneous switching in ambiguous situations may reflect dispositional differences in the proneness to cognitive flexibility (Armbruster et al. 2012), we hypothesized that higher rates of spontaneous switching on ambiguous trials should be associated with higher self-reported resilience (Hypothesis 2). Third, can we replicate the finding of Genet & Siemer (2011) that cognitive and affective flexibility are unique predictors of resilience (Hypothesis 3)? Fourth, how are switch costs in the affective flexibility paradigm influenced by the task to be performed and the valence of the stimuli before and during switching? We expected a main effect of task on switch costs, independent of stimulus valence, which would replicate Reeck & Egner’s (2015) result of higher switch costs when switching to the emotion task as compared to switching to the neutral gender task (Hypothesis 4a). Based on the results of an unpublished pilot study conducted in an independent group of participants (which aimed to assure the feasibility of the paradigm with respect to content, length, and pace of the task), we furthermore predicted an interaction between task and valence of the stimulus before the switch trial (Hypothesis 4b) as well as between task and valence of the current stimulus on which the task switch is being conducted (Hypothesis 4c). Lastly, we hypothesized that more resilient persons should be better able to disengage from negative or aversive experiences, so that we predicted an association between higher resilience scores and lower switch costs particularly when switching from negative to positive stimuli (Hypothesis 5).

## Methods

### Participants

A sample of *N* = 100 students (from subject areas other than psychology) were included in the study (50 females, age 18–35, mean age 23.7 ± 3.8 years). Sample size was based on an a priori power analysis in G*power 3.1 (Faul et al. 2007) for detecting small to moderate effects in a one-sided correlation analysis (*r* = 0.25, *p* = 0.05, power = 0.80; based on the correlation reported in Genet & Siemer, 2011), which resulted in a minimum sample size of *N* = 97. In the pre-registered analysis plan, it was intended to exclude all subjects with error rates of 30% or higher in any of the acquired experimental conditions. However, it was found that in the case of the cognitive flexibility paradigm with a total of four conditions, this applied to almost one-fifth of the participants. Therefore, we decided to exclude subjects only from analyses involving those experimental condition in which they showed error rates of 30% or higher and to include them in all other analyses. Final sample sizes, accordingly, varied between analyses (*N* = 83 in case of ambiguous trials and *N* = 91 to 100 in the other conditions) and are reported together with the respective results. Participants provided written informed consent according to procedures approved by the Ethics Committee of the Department of Psychology of Goethe University, Frankfurt, Germany.

### Procedure

Participants first received a link to a survey generated in the online tool unipark (Questback GmbH, Cologne, Germany) to fill in the three resilience questionnaires. Results of the survey were saved anonymously using an identification code. After completing the survey, participants received a confirmation of participation with the identification code, which they were asked to bring to their appointment at the Department of Psychology, where they performed the experimental part of the study. Prior to the actual experiments, participants completed several training blocks to get familiar with the tasks. The order of the experiments was counterbalanced across subjects.

### Questionnaires

Windle and colleagues (2011) compared 15 published self-rating scales for measuring resilience regarding their psychometric properties, but found no ‘gold standard’ amongst these measures. Three scales received the best ratings in this comparison – the Connor-Davidson Resilience Scale (CD-RISC; Connor & Davidson 2003), the Brief Resilience Scale (BRS; Smith et al. 2008), and the Resilience Scale for Adults (RSA; Friborg et al. 2005). The last-mentioned scale, RSA, assesses both intrapersonal and interpersonal protective mechanisms by including scales for social support and family coherence. The other two scales — like most self-report measures of resilience – focus on characteristics of the individual person: The BRS follows an outcome-oriented approach measuring “the ability to bounce back or recover from stress” (Smith et al. 2008, p. 194), while the CD-RISC defines resilience as a personality trait by assessing “personal qualities that enable one to thrive in the face of adversity” (Connor & Davidson 2003, p. 76). In the present study, we administered the CD-RISC (German version by Sarubin et al. 2015) and the BRS (German version by Chmitorz et al. 2018). In addition, the Resilience Scale (RS; Wagnild & Young 1993; German version by Schumacher et al. 2005) as another trait-oriented questionnaire was included as it is relatively widely used in the relevant literature. For each of the three questionnaires a resilience score was calculated according to the respective instruction manuals. As additional post-hoc exploratory analyses, we calculated a composite score for the three resilience scores by z-standardizing and summing them up and then repeated the correlations between resilience and flexibility using this single resilience score.

### Experimental tasks

Cognitive and affective flexibility were assessed in two separate experimental paradigms. Both paradigms were designed to ensure an unambiguous association between response and task (by using four different response keys), thereby avoiding ambiguity concerning which task the participant intends to perform (compare Genet & Siemer 2011) and thus also allowing us to consider error rates as an additional outcome measure.

**Fig. 1:**
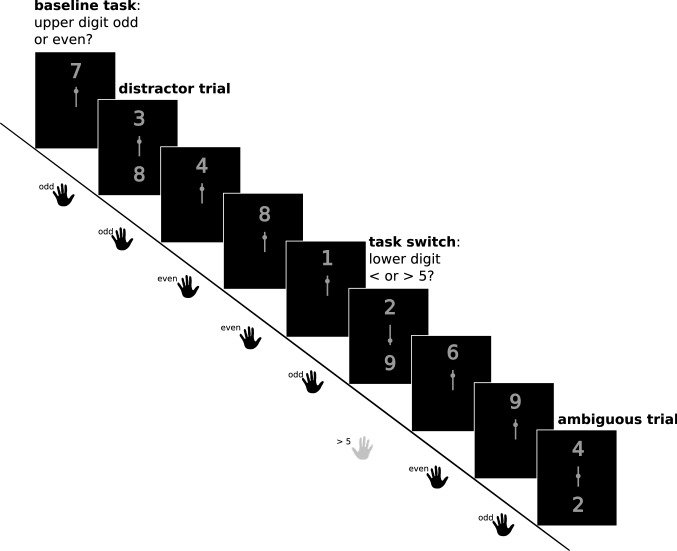
Illustration of the cognitive flexibility task. Baseline task: Baseline or ongoing task trials present only one stimulus above the central cue and require an odd vs. even discrimination to this stimulus. Distractor inhibition trials: Two digits are presented simultaneously. The cue in the middle of the screen indicates to ignore the lower digit and respond to the upper one (odd/even discrimination). Task switch trials: Two digits are presented and the cue indicates to ignore the upper digit and respond to the lower stimulus with a < / > 5 discrimination judgment with the other hand. Ambiguous trials: Two digits are presented, but the cue does not clearly indicate which digit to respond to. This figure was originally published in Kraft et al. (2020) under a CC—BY 4.0 license

For cognitive flexibility, an adaptation of a paradigm developed by Armbruster and colleagues (2012) was used which involves the same task rules as the task switching paradigm reported in the study by Genet & Siemer (2011), i.e., judgments of parity (odd vs. even) and magnitude (greater vs. lower than five), applied to single digits between ‘1’ and ‘9’ (excluding ‘5’). However, in contrast to the paradigm by Genet & Siemer (2011), the task rule was not indicated by a simultaneously presented cue word but by the position of the digit on the screen in combination with a symbol in the middle of the screen (see Fig. 1 for a visualization of the experimental task): In 80% of trials (‘baseline’ or ‘ongoing’ condition), a single digit was presented in the upper part of the screen for one second in light grey on a black background and participants were required to perform an odd/even discrimination. Responses were given by pressing two buttons with the index and middle finger of the left hand. However, in 20% of the trials a second digit appeared in the lower half of the screen and a cue in the middle indicated which digit the participants should respond to. The cue consisted of a vertical line, on which a small dot was shown at variable locations (Fig. 1). If the cue indicated the upper digit (i.e., if the dot was located closer to the upper than to the lower digit), participants had to ignore the lower digit and to respond to the upper digit as before (distractor inhibition or ‘cognitive stability’ condition; 1/3rd of critical trials). If, however, the lower digit was cued, participants were instructed to switch to the lower digit and respond to the other task rule (i.e., greater/lower 5) by pressing a button with either the index or middle finger of the right hand (switch or ‘cognitive flexibility’ condition; 1/3rd of critical trials). Last, in the remaining third of trials, the cue was ambiguous, i.e., it did not indicate a clear preference concerning which digit the participants should respond to. This ambiguous condition was used to assess the rate of spontaneous switching, i.e. an individual disposition to show flexible behavior in the presence of equivocal information (see Armbruster et al. 2012, for more details). After each critical trial (i.e., distractor inhibition, switch, or ambiguous condition), participants continued to perform the baseline task (upper digit). Each stimulus was followed by the presentation of a fixation cross for 1 second, resulting in a trial duration of 2 seconds (which also defined the maximum possible response time). The paradigm comprised two blocks of 150 trials, resulting in a total experiment duration of approximately ten minutes (excluding the variable duration of the break between the blocks). The baseline condition, accordingly, involved 240 trials, while each of the three critical conditions involved 20 trials.

**Fig. 2:**
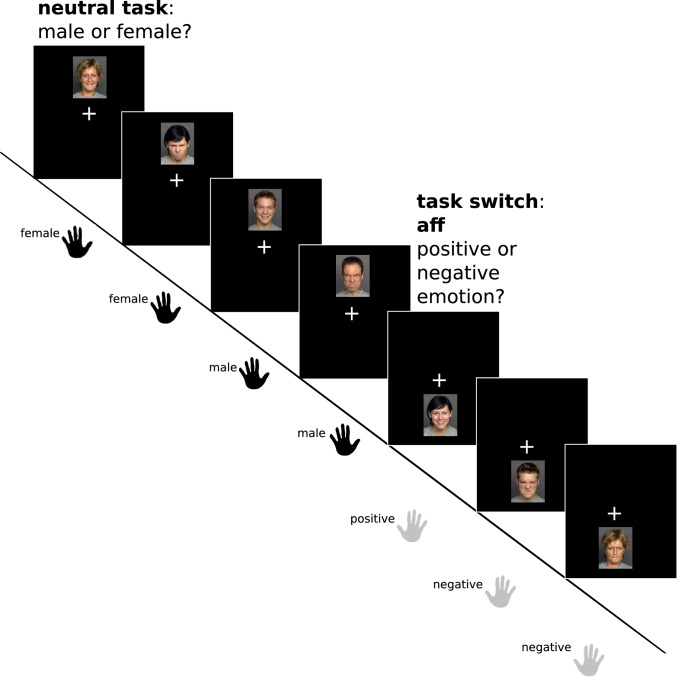
Illustration of the affective flexibility task. Pictures in the upper part of the screen require a gender classification (male vs. female). For pictures in the lower part of the screen participants classify the emotional facial expression (positive or negative). Task switches (i.e., changes of the location of the images) occur every 3–7 trials and are always accompanied by a switch of the response hand. This figure was originally published in Kraft et al. (2020) under a CC—BY 4.0 license

Affective flexibility was examined with a paradigm that combines aspects of the cognitive flexibility paradigm described above with the affective task switching paradigm reported by Dierolf et al. (2016). In contrast to the paradigm used by Genet & Siemer (2011), pictures of emotional faces are used as affective stimuli instead of positive/negative words. Pictures of 120 different faces were selected from the FACES database (Ebner et al. 2010) according to how accurately the angry expression had been identified by a sample of *N *= 154 raters (Ebner et al. 2010): The images of 60 female and 60 male persons with lowest error rates for the emotion ‘angry’ were used for both the angry and the happy condition, resulting in a total of 240 stimuli. Participants switched between a gender classification and a valence classification task, whereby the to-be-performed task was specified by the stimulus location on the screen (see Fig. 2): Whenever pictures appeared in the upper part of the screen, participants were instructed to indicate whether the depicted person was female or male by pressing a button with the left index or middle finger; for pictures in the lower part of the screen participants indicated whether the person showed a positive or negative emotional expression, by a button press with the right hand. After a task switch, the new task continued for two to six more trials until the next task switch occurred. Critical comparisons thus involve switch versus repeat trials. To reduce complexity and to obtain sufficiently large trial numbers that allowed us to investigate valence effects on switch costs, there were no distractor or ambiguous conditions in this paradigm. Total length was approximately 8 min and the experiment consisted of 240 trials of two seconds length (1 s picture, 1 s fixation cross, maximum response time 2 s). In total, each participant performed 48 switch trials and 192 repetition trials, and switch trials were balanced so that the number of switches from the neutral to the affective task and vice versa, as well as switches from happy to angry faces and vice versa, were equated.

A previous study has shown excellent internal consistency and good test –retest reliability for response time switch costs in this task, but substantially lower reliability estimates for error rate-based switch costs (Eckart et al. 2021).

### Data analyses

Data analysis was performed in Python 3 (Version 3.6.9; www.python.org) using the pandas, pingouin, scipy, numpy, statsmodels, and seaborn packages. Following the preregistered analysis plan, all trials with response times below 250 ms or greater than three standard deviations above the person’s mean of the respective condition were considered outliers and were thus excluded from the analyses. The following indices were then calculated: For the cognitive flexibility paradigm, ‘costs’ in terms of response times and errors were calculated for correct trials in all three critical conditions, i.e., switching, distractor inhibition, and ambiguous trials, relative to the baseline condition (note that ambiguous trials were considered correct depending on the combination of response and response hand, as the two tasks were assigned to different hands). Furthermore, we determined the ‘spontaneous switch rate’, i.e., the individual percentage of switches in ambiguous trials. For the affective flexibility paradigm, switch costs (i.e., response times and errors in switch relative to repetition trials performed on the same task) were calculated separately for switches to the emotion task and for switches to the gender task. In order to analyze effects of affective valence of the stimuli, switch costs were also calculated for all eight possible combinations of task and facial expressions before and during switching (e.g., switching from a happy face in the gender task to an angry face in the emotion task etc.).

To test our hypotheses concerning the relationship between indices of flexibility (as well as stability) and resilience, one-sided Spearman correlations (*r*_*s*_) were calculated between resilience scores on the one hand and switch costs, distractor inhibition costs, and the spontaneous switch rate of the cognitive paradigm, as well as switch costs elicited when switching to the gender or to the emotion task in the affective paradigm. To correct for multiple testing, Dubey–Armitage–Parmar correction was applied which accounted for intercorrelations of the three measures of resilience (Sankoh et al. 1997). Correlations were assessed using one-sided statistical tests, as our hypotheses clearly specified the direction of expected correlation. Critical p-values after correction for three endpoints were *p* = 0.037 for correlations with the CD-RISC or RS-25 and *p* = 0.034 for correlations with the BRS (note that different p thresholds reflect the different correlation strengths between the three questionnaires). In order to test whether flexibility is differentially associated with trait- vs. outcome-related measures of resilience (exploratory analysis associated with Hypothesis 1), statistical comparisons between (a) the correlations of trait-based resilience (i.e., CD-RISC and RS) with (cognitive and/or affective) switch costs, and (b) the correlations of outcome-based resilience scores (i.e., BRS) with switch costs were calculated with the software ‘cocor’ (Diedenhofen & Musch, 2015). To examine whether cognitive and affective flexibility account for unique variance components in self-reported resilience, multiple regression analyses with resilience scores as dependent variables and cognitive and affective switch costs as predictors were performed (separately for response times and error rates, and separately for the different resilience scores). Lastly, to examine how switch costs in the affective flexibility paradigm are influenced by the valence of the stimuli before and during switching, analyses of variance (ANOVA) were performed for switch costs (separate analyses for response times and errors) with within-subject factors ‘task’ (task to which participants are switching; Hypothesis 4a), ‘valence before switching’ (Hypothesis 4b), and ‘valence of switch trial’ (Hypothesis 4c). In addition, one-sided Spearman correlation tests were calculated between resilience scores and switch costs in trials involving switches away from negative stimuli and toward positive information (separately for response times and errors) to test the hypothesis that more resilient persons should be better able to disengage from negative or aversive experiences and turn attention to positive information (Hypothesis 5).

## Results

### Descriptive statistics

#### Questionnaires

The mean and range of resilience scores for the three questionnaires can be found in Table 1. Compared to the samples of published validation studies of the three questionnaires, our sample shows slightly lower resilience scores (for comparison: German version of the CD-RISC, 73.3 for men and 74.4 for women, Sarubin et al. 2015; German version of the RS-25, 136.6 for men and 134.1 for women, Schumacher et al. 2005; German version of the BRS, 3.58 and 3.37 in two samples, Chmitorz et al. 2018). The three resilience measures were highly intercorrelated (CD-RISC and RS-25: *r*_*s*_ = 0.80, *p* < 0.001; CD-RISC and BRS: *r*_*s*_ = 0.65, *p* < 0.001; RS-25 and BRS: *r*_*s*_ = 0.61, *p* < 0.001).

**Table 1:** Descriptive statistics of resilience questionnaires (*N* = 100)

Resilience scores	Mean (SD)	Range (possible range)	Cronbach’s α
CD-RISC	66.5 (13.3)	30–93 (25–125)	0.89
RS-25	130.7 (19.6)	74–168 (25–175)	0.90
BRS	3.3 (0.8)	1.2–5.0 (1–5)	0.85

*SD* standard deviation, *CD-RISC* Connor-Davidson Resilience Scale, *RS-25* Resilience Scale, *BRS* Brief Resilience Scale. See Methods section for references.

#### Flexibility indices

Excluding subjects with error rates higher than 30 percent in either the gender or emotion task (see Methods) resulted in a sample of *N* = 99 subjects for the affective flexibility paradigm. For the cognitive flexibility paradigm, sample size decreased to *N* = 94 in the switch condition, *N* = 91 in distractor inhibition trials, and *N* = 83 in the ambiguous condition after exclusion of subjects with error rates of 30% or higher in the respective experimental condition (see Methods). Mean response times and percentages of errors for each condition of the cognitive and affective flexibility paradigms can be found in Table 2. As reported in previous research (Armbruster et al. 2012), response times in the cognitive flexibility task were fastest in baseline trials. Responses in distractor inhibition trials were slowed relative to baseline, but faster than in switch trials, and responses were slowest in ambiguous trials. Mean switch costs (calculated for each subject as the difference between mean response times during switching and the baseline condition) were 343 ms, varying between 120 and 725 ms across subjects (SD = 130, *N* = 94). Mean distractor inhibition costs were 195 ms (SD = 112, range 12–686 ms, *N* = 91). The mean spontaneous switch rate (percentage of switches in ambiguous trials) was 46%, ranging from 0 to 100% (SD = 32, *N* = 83). Response time switch costs in the affective flexibility paradigm (calculated as the difference between mean response times during switch and repetition trials of the respective condition, *N* = 99) were 246 ms (SD = 97, range 39–536 ms) for switches from the emotion to the gender task and 270 ms (SD = 119, range 84–627 ms) for switches from the gender to the emotion task. All response time cost indices were significantly different from zero, as determined using t tests (all *p *< 0.001).^1^ Internal consistency estimates ranged between 0.82 and 0.93 for response time switch costs and between 0.37 and 0.62 for error rate switch costs (for details see Kraft et al. 2020).

**Table 2:** Response times and error rates in the two experimental paradigms

Condition	Response times (ms) Mean (SD)	Errors (%) Mean (SD)
*Cognitive flexibility*		
Baseline trials (*N* = 100)	688 (88)	4.5 (3.1)
Distractor trials (*N* = 91)	886 (155)	8.3 (8.2)
Switch trials (*N* = 94)	1,033 (165)	8.8 (8.8)
Ambiguous trials (*N* = 83)	1,140 (205)	15.3 (9.4)
*Affective flexibility (N* = *99)*		
Baseline gender task	655 (92)	5.5 (3.7)
Baseline emotion task	709 (89)	7.5 (5.5)
Switches emotion → gender	901 (141)	6.9 (8.5)
Switches gender → emotion	979 (161)	15.8 (14.7)

*SD*  standard deviation. Sample sizes vary between experimental conditions as subjects with error rates of 30% or higher were excluded from analyses of the respective condition.

Analogous to response times, error rates in the cognitive flexibility task were also lowest in the baseline condition and highest in ambiguous trials. However, in contrast to response times, error rates were highly similar between distractor inhibition and switch trials: Error switch costs (calculated as the difference between switching and the baseline condition) were 4.6%, varying between -10% and + 23% across subjects (SD = 7.8, *N* = 94). Mean error distractor inhibition costs were 4.0% (SD = 6.9, range between − 9 and 24%). Error switch costs in the affective flexibility paradigm (*N* = 99) were 1.4% (SD = 7.3, range – 13 to + 41%) for switches from the emotion to the gender task and 8.2% (SD = 11.9, range − 10 to 53%) for switches from the gender to the emotion task. All error cost indices differed significantly from zero (*p* < 0.001) except the costs when switching from the emotion to the gender task (*p* = 0.06).^1^

### Correlation of resilience and flexibility measures (Hypotheses 1 and 2*)*

To examine the relationship between self-rated resilience and psychological flexibility, Spearman correlations between resilience scores and (response time as well as error) switch costs derived from the cognitive and affective flexibility paradigms were calculated. As expected (Hypothesis (1), response time switch costs of the affective paradigm were negatively correlated to all three self-report measures of resilience (see Table 3), such that lower affective switch costs were associated with higher resilience scores. This correlation was statistically significant (taking into account correction for multiple comparisons; see Methods) for all three questionnaires for switches from the gender to the emotion task, and for two out of three resilience questionnaires (CD-RISC, RS-25) for switches from emotion to the neutral task. For the cognitive flexibility paradigm, there was also a negative correlation between response time switch costs and resilience, but only for one of the three questionnaires, the CD-RISC (see Table 3 for statistics). Comparisons of correlations were calculated to test whether flexibility is differentially associated with trait- vs. outcome-related measures of resilience (as measured using CD-RISC and RS-25 vs. BRS scales, respectively). There were, however, no significant differences between the correlations of (cognitive or affective) switch costs with CD-RISC or RS-25 vs. BRS (all *p* between 0.32 and 0.72). An exploratory analysis using a composite score from the three questionnaires yielded significant correlations with switch costs of both paradigms (see Supplemental Material S1).

**Table 3:** Correlations of response time-based measures of flexibility/stability and resilience questionnaires

	*CD-RISC*	*RS-25*	*BRS*
*Affective flexibility task (N* = *99)*			
Switch costs emotion → gender	***r***_***s***_** = − 0.23, *p* = 0.01**	***r***_***s***_** = − 0.22, *p* = 0.01**	*r*_***s***_ = − 0.15, *p* = 0.07
Switch costs gender → emotion	***r***_***s***_** = − 0.31, *p* = 0.001**	***r***_***s***_** = − 0.30, *p* = 0.001**	***r***_***s***_** = − 0.23 *p* = 0.01**
*Cognitive flexibility task*			
Switch costs (*N* = 94)	***r***_***s***_** = − 0.20, *p* = 0.03**	*r*_*s*_ = **− **0.13, *p* = 0.11	*r*_***s***_ = **− **0.17, *p* = 0.047
Distractor inhibition costs (*N* = 91)	*r*_***s***_ = 0.11, *p* = 0.16	*r*_***s***_ = 0.07, *p* = 0.25	*r*_***s***_ = 0.01, *p* = 0.45
Spontaneous switching (*N* = 83)	*r*_***s***_ = 0.06, *p* = 0.29	*r*_***s***_ = **− **0.03, *p* = 0.40	*r*_***s***_ = **− **0.08, *p* = 0.23

All tests are one-sided. Statistically significant results (based on critical *p*-values determined using Dubey–Armitage–Parmar correction for three endpoints; see Methods section for details) are printed in bold. CD-RISC = Connor-Davidson Resilience Scale; RS-25 = Resilience Scale; BRS = Brief Resilience Scale.

In contrast to our supplementary Hypothesis 1a, error rate switch costs did not correlate with resilience scores (all *p* between 0.11 and 0.50). Consistent with Hypothesis 1b, our data provided no evidence for an association between cognitive stability (i.e., distractor inhibition costs) and self-reported resilience (all *p* between 0.16 and 0.45). We also found no support for Hypothesis 2, as the spontaneous switch rate did not correlate with resilience scores (all *p* between 0.23 and 0.40).

### Predicting resilience from cognitive and affective flexibility: regression analyses (Hypothesis 3)

Multiple regression analyses with resilience scores as dependent variables and cognitive and affective switch costs as predictors were computed to test whether cognitive and affective flexibility account for unique variance components of self-reported resilience. In contrast to Hypothesis 3 and a previous report by Genet & Siemer (2011), only affective RT switch costs, but not cognitive RT switch costs, were a predictor of resilience. This became significant for the CD-RISC and RS-25, but not for the BRS (see Table 4). The regression analyses for error rate switch costs showed a significant result only for the prediction of CD-RISC (*R*^2^ = 0.065, *F*(2,90) = 3.147, *p* = 0.048) and again only for affective switch costs (*β* = − 0.259, *p* = 0.016*)* but not for cognitive switch costs (*β* = 0.014, *p* = 0.89*)*.

**Table 4:** Results of simultaneous regression analysis for response time switch costs predicting resilience questionnaires

	*β*	*p*
*CD-RISC (R*^*2*^ = *0.101, F(2,90)* = *5.03, p* = *0.009)*		
Cognitive switch costs	-0.005	0.686
Affective switch costs	**− 0.039**	**0.020**
*RS-25 (R*^*2*^ = *0.098, F(2,90)* = *4.86, p* = *0.01)*		
Cognitive switch costs	− 0.002	0.910
Affective switch costs	**− 0.062**	**0.014**
*BRS (R*^*2*^ = *0.079, F(2,90)* = *3.85, p* = *0.025)*		
Cognitive switch costs	− 0.000	0.605
Affective switch costs	− 0.002	0.053

Statistically significant results are printed in bold

*CD-RISC* Connor-Davidson Resilience Scale, *RS-25* Resilience Scale, *BRS* Brief Resilience Scale

### Valence effects on affective flexibility (Hypotheses 4 and 5)

To examine how switch costs in the affective flexibility paradigm are influenced by the valence of the stimuli presented before and during task switching, 2 × 2 × 2 analyses of variance were performed with switch costs as dependent variables using the within-subjects factors ‘task’ (emotion vs. gender), ‘valence before switching’, and ‘valence of switch trial’. Four persons were found not to have scored a single hit in at least one of the eight switch conditions in this analysis,^2^ which is why they had a missing value for reaction time of that condition and were not included into the ANOVA, resulting in *N* = 95 for this analysis. A main effect of ‘task’ was found for response time switch costs (F(1,94) = 8.96, *p* = 0.004) as expected (Hypothesis 4a), with higher switch costs when switching from the gender to the emotion task (mean: 267 ms) than vice versa (243 ms). In addition, a main effect of ‘valence of switch trial’ (F(1,94) = 4.88, *p* = 0.03) revealed higher switch costs when switching to a happy face (259 ms) than to an angry face (248 ms), and a main effect of ‘valence before switching’ (F(1,94) = 4.40, *p* = 0.04) reflected higher switch costs when the preceding picture showed an angry face (259 ms) compared to a happy face (248 ms).

**Fig. 3:**
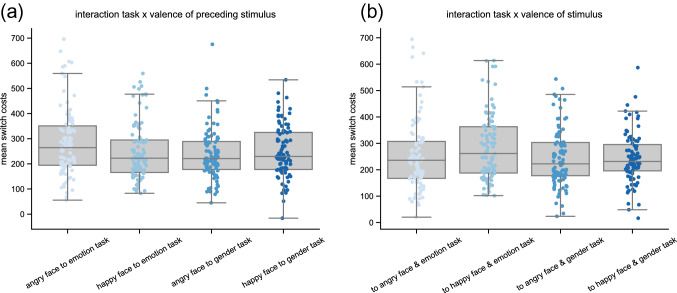
Valence effects on response time switch costs **a** Significant interaction between the valence of the preceding stimulus and the task of the switch trial. **b** Significant interaction of the valence of the stimulus presented in the switch trial and the task. Black line represents the median, grey boxes reflect the 1st quartile and 3rd quartile, and whiskers show 1.5 times the interquartile range from the lower and upper bounds of the box

In line with our Hypothesis 4b, there was a significant interaction between ‘valence before switching’ and ‘task’ (F(1,94) = 15.71, *p* < 0.001, see Fig. 3a): When switching away from an angry face to the emotion task, reaction time costs were higher (285 ms) than when switching away from a happy face (249 ms; *t(94)* = 4.37; *p* < 0.001); when switching to the gender task there was no significant difference (249 ms for happy faces before switch, 237 ms for angry faces before switch; *t(94)* = − 1.36; *p* = 0.18). (Note, however, that Hypothesis 4b did not further specify the direction of this interaction.) Furthermore, as hypothesized (Hypothesis 4c), there was also a significant interaction of ‘task’ and ‘valence of switch trial’ (F(1,94) = 5.30, *p* = 0.02, see Fig. 3b). Post-hoc t-tests showed that switch costs did not differ between switch trials with happy (244 ms) vs. angry faces (244 ms) when switching to the gender task (*t(94)* = − 0.00; *p* = 0.997), but that switch costs were significantly longer for happy (284 ms) than for angry faces (255 ms) when switching to the emotion task (*t(94)* = − 2.69; *p* = 0.008). Note also here that Hypothesis 4c did not specify the direction of the interaction effect. Lastly, there was no interaction of ‘valence before switching’ with ‘valence of switch trial’ and also no significant three-way interaction.

The ANOVA calculated on error rate switch costs also revealed a main effect of ‘task’ (F(1,98) = 27.48, *p* < 0.001) with higher switch costs when switching from the gender to the emotion task (8.2 percentage points increase in error rate) than vice versa (1.4 percentage points), which is consistent with Hypothesis 4a. In analogy to response time switch costs, a main effect of ‘valence of switch trial’ was also found (F(1,98) = 7.51, *p* = 0.007), reflecting higher error rate switch costs when switching to a happy face (6.5 percentage points) than to an angry face (3.1 percentage points). There was no significant main effect of ‘valence before switching’ or interaction effect.

To test the hypothesis that more resilient persons should be better able to disengage from negative experiences and turn attention to positive information (Hypothesis 5), correlations were calculated between resilience scores and switch costs in trials involving switches away from angry faces and toward happy faces. There was no consistent relationship between resilience measures and error rate switch costs when switching away from angry faces toward happy faces (*p-values* between 0.06 and 0.24). For RT switch costs, a significant negative correlation was found with resilience scores of the CD-RISC (*r*_*s*_ = − 0.20, *p* = 0.03), while for the other two questionnaires, correlations did not reach corrected significance thresholds (RS-25: *r*_*s*_ = − 0.16, *p* = 0.05; BRS: *r*_*s*_ = − 0.18, *p* = 0.04; critical *p*-values *p* = 0.037 and *p* = 0.034, respectively; see Methods section for details on multiple comparison correction). However, exploratory (i.e., not pre-registered) analyses showed that the correlation with CD-RISC was not specific for switches from angry to happy faces: In fact, costs elicited when switching in the opposite direction (from a happy face to an angry face) correlated highly significantly with resilience scores of all three questionnaires (CD-RISC: *r*_*s*_ = − 0.34, *p* < 0.001; RS-25: *r*_*s*_ = − 0.28, *p* = 0.003; BRS: *r*_*s*_ = − 0.30, *p* = 0.001; see Supplemental Material S2).

### Covariates

According to our pre-registered analysis plan, gender, age, handedness, drug use, and history of psychiatric disorder were considered as possible confounding variables. Results of these analyses can be found in the Supplemental Material S3.

## Discussion

The present study examined task switching costs to investigate (i) whether cognitive or affective flexibility are related to self-reported resilience, thereby differentiating between trait- and outcome-oriented definitions of resilience, (ii) whether cognitive and affective flexibility are unique predictors of resilience, and (iii) whether a relationship with resilience can also be found for individual differences in the proneness to cognitive flexibility operationalized as spontaneous switching in ambiguous situations. In addition, we explored (iv) whether the efficiency of affective task switching is influenced by the valence of stimuli and (v) whether the valence of stimuli affects the relationship between affective flexibility and resilience. As already previously reported for this dataset (Kraft et al. 2020), robust switch cost effects occurred in both experimental paradigms, replicating earlier work (Armbruster et al. 2012; Eckart et al., 2021a; Ueltzhöffer et al. 2015). In line with previous research (Genet & Siemer, 2011), subjects with higher resilience scores were more efficient (in terms of RT switch costs) in affective task switching. The same relationship was found for cognitive flexibility, but only for one of the three resilience questionnaires (CD-RISC). In contrast, measures of cognitive stability and spontaneous switching in ambiguous situations did not correlate with self-reported resilience. Regression analyses indicate that affective but not cognitive flexibility accounted for unique variance components in self-reported resilience. Regarding possible effects of stimulus valence, we found that RT switch costs were influenced (as reflected in main and interaction effects) by the task (affective vs. non-affective), the valence of the stimulus before switching, and the valence of the switch trial. However, our data do not support that the correlation between resilience and affective flexibility is limited to switches between stimuli of certain valence. In the following, we will discuss results concerning affective task switching, before we consider in more depth the association between resilience and affective and cognitive flexibility.

### Affective task switching

As expected (Hypothesis 4a) and in analogy to previous findings (Aboulafia-Brakha et al. 2016; Eckart et al. 2021; Reeck & Egner, 2015), asymmetric switch costs were observed in the affective flexibility task: switching to the emotion task resulted in higher switch costs (longer RT and more errors) than switching to the gender task. Generally, successful task switching requires suppression of the previous task while the currently relevant task set is activated. If the two task rules differ in their relative dominance, task switches to the dominant task are usually more costly, as stronger backward inhibition has to be overcome to re-activate the task (i.e., negative priming effects; Monsell et al. 2000; Wylie & Allport, 2000). Given that affective materials typically capture attention more strongly (e.g., Niu 2012), it seems reasonable to assume that switch cost differences between the two tasks rely on the affective valence judgement being more dominant than affectively neutral gender judgements. A complementary explanation attributes asymmetric switch costs to an acceleration of response times in the less dominant task. The higher degree of cognitive control, which is expended for the weaker task to ensure its proper performance, should result in generally improved performance in this task (Yeung & Monsell 2003). Indeed, task-switching between affective and non-affective task rules has in previous work been characterized by response accelerations in the non-affective task, instead of response decelerations in the affective task (Schuch et al. 2012). However, Yeung & Monsell (2003) propose that both mechanisms, priming and endogenous cognitive control can influence task switching, so that these two possible explanations do not have to be mutually exclusive.

Furthermore, a significant influence of stimulus valence on affective task switching was revealed. Threat-related stimuli are evolutionarily significant and capture attention via stimulus-driven bottom-up processes (e.g., Engen et al. 2017; Öhman et al. 2001, 2012). In line with this assumption, the presence of threat-related information (i.e., angry face) accelerated task switching, while threat-related information on the pre-switch trial slowed it down. While the former effect is presumably due to automatic attention capture by the evolutionarily significant stimulus, the latter effect might be attributed to increased backward inhibition that is necessary to actively draw attention away from the threat-related stimulus. Interestingly, these effects were only present in the emotion task, i.e., when threat-related information was task-relevant. This result might also be related to the possibility that increased cognitive control is expended for the less dominant gender task (Yeung & Monsell 2003 see previous paragraph) as increased top-down control might overrule stimulus-driven, bottom-up effects of threat-related information (and abolish them in the less dominant task).

### Affective flexibility and resilience

The present study replicates the finding of Genet & Siemer (2011) that persons with higher resilience scores were more efficient in affective task switching when considering response time switch costs. We could show this effect for three different resilience questionnaires and for both switch directions: Correlations were statistically significant for all three questionnaires for switches from the gender to the emotion task, and for two out of three questionnaires (CD-RISC, RS-25) for switches from emotion to the neutral task (the correlation with BRS also pointed in the same direction but missed significance with *p* = 0.07). Thus, the present data support our Hypothesis 1 and suggest that the relationship between affective response time switch costs and resilience is a robust effect that is evident across different measures of resilience.

In this context, we had hypothesized that associations with resilience would show up particularly when switching away from negative stimuli and to positive stimuli, as more resilient persons should be better able to disengage from negative or aversive experiences (Hypothesis 5). In line with this hypothesis, we detected a significant negative correlation between these response time switch costs and resilience scores of the CD-RISC, but correlations did not reach significance for the other two questionnaires. Importantly, however, exploratory analyses showed that the correlation with CD-RISC scores was not specific to costs when switching from negative to positive information. On the contrary, there were also highly significant correlations between the resilience scores of all three questionnaires and switch costs of the reverse valence direction (switch from positive to negative affect). While this latter finding is in line with the results of Grol & De Raedt (2018), who reported a marginally significant relationship between resilience and more efficient task switching (in a slightly smaller sample) when a negative stimulus was preceded by a positive one, the robust correlations independent of switch direction observed in the present study speak against a specific role of flexible disengagement from aversive information for resilience. We thus interpret this result as lack of support for our Hypothesis 5. Beyond that, it must be acknowledged that across studies, a rather heterogenous pattern of associations between affective task switching and markers of individual differences in affective dimensions emerges: For example, affective task switching predicted individual differences in rumination (Genet et al. 2013), reappraisal (Malooly et al. 2013), and future anxiety and worries (Twivy et al. 2020), but differently for negative as opposed to positive stimuli and inconsistently across studies. We thus propose that further research is needed and that replication of previous results should be among the first research goals.

Distinct from the results for RT switch costs, no reliable correlations were found between error rate switch costs and resilience measures (Hypothesis 1a). This seems to be consistent with the existing literature: Genet & Siemer (2011) and Hildebrandt et al. (2016) reported no error rate costs for switching and limited their analyses to response times. Grol & De Raedt (2018) analyzed error rates (but not switch costs) and found only a correlation with a subscale of the Resilience Scale and only when controlling for the change of positive mood during their mood induction paradigm. The absence of robust correlation effects for error rate switch costs is furthermore consistent with our recent finding that error rate switch costs in the affective domain have substantially lower psychometric reliability (both in terms of test retest as well as internal consistency; Eckart et al. 2021) than response time switch costs, so that error rate switch costs might simply not be well-suited to measure individual differences (see Eckart et al. 2021, for an in-depth discussion).

### Cognitive flexibility and resilience

For the cognitive flexibility paradigm, we found an association with response time switch costs only for one of the three resilience questionnaires, the CD-RISC. For the other two questionnaire measures, the correlations pointed in the same direction, but were not significant (*p* = 0.11 and 0.05). Thus, we replicate the correlative results of Genet & Siemer (2011), who also worked with the CD-RISC questionnaire, but could not extend them to other self-report measures. We consider this partial support for our Hypothesis 1 with respect to cognitive flexibility.

Most importantly, however, our regression results for cognitive flexibility are not fully consistent with those of Genet & Siemer (2011): When simultaneously including affective and cognitive switch costs as predictors of resilience, only affective but not cognitive RT switch costs were a significant predictor of resilience. Our data, thus, provide no evidence for independent contributions of cognitive and affective flexibility to resilience, and Hypothesis 3 (mainly derived from the work of Genet & Siemer, 2011) cannot be supported. In this context, it is also important to consider that cognitive and affective switch costs did not correlate in the study by Genet and Siemer (2011) – and also showed a heterogenous pattern of inter-correlations across other studies, often depending on valence (Genet et al. 2013; Malooly et al. 2013) – whereas our data show robust correlations between affective and cognitive (non-affective) switch costs (see Kraft et al. 2020 for in-depth analysis). This difference may be related to methodological differences between studies, like the fact that Genet & Siemer (2011) used different stimuli and only two response buttons, so that the correct response for the relevant task sometimes was mapped on the same button as the correct response for the non-active task (in consistent task blocks) and sometimes not, which may have introduced interference effects. Our data do not allow us to resolve the effects of such procedural differences. However, the observed multicollinearity between predictors may have hampered our ability to isolate unique contributions in the prediction model. On the other hand, the correlation between affective and cognitive switch costs may reflect shared underlying cognitive mechanisms (Kraft et al. 2020) – which would also plausibly explain the absence of unique prediction for both switch cost markers.

As a new variable, the rate of spontaneous switching in ambiguous situations was included in the current study as a potential measure of dispositional differences between persons in the proneness to switch tasks (Armbruster et al. 2012). However, in contrast to our expectation (Hypothesis 2), spontaneous switching did not correlate with resilience scores. This finding could indicate that it is the efficiency of switching (indicated by RT switch costs) rather than the proneness of switching per se, which is related to resilience. Note, however, also that the spontaneous switch rate is still not yet fully understood as a marker for cognitive flexibility and future research is needed to clarify its role. For example, it is conceivable that spontaneous switching can be influenced by factors other than dispositional differences in the proneness to cognitive flexibility, such as effort avoidance or boredom during the task, which would reduce or increase, respectively, the proneness to switch (e.g., Geana et al. 2016; Kool et al. 2010). For this reason, we consider at present the results for switch costs more promising for understanding the relationship between resilience and different facets of psychological flexibility. Last, our results also show that cognitive stability, measured here as the RT or error rate costs of inhibiting a task-irrelevant distractor, is not associated with resilience (Hypothesis 1b).

### Flexibility and trait- vs. outcome-oriented resilience

The three questionnaire measures of resilience studied here have been identified as having the best psychometric properties in a comparative review (Windle et al. 2011). CD-RISC/RS-25 and BRS differ in that they define resilience as trait or outcome, respectively, i.e., the former two concentrate on (personality) traits that are considered favorable in facing adverse life events, while the latter one measures the perceived ability to recover after having experienced these events. We consider it important to include both conceptualizations when studying resilience. However, in the present work, all three questionnaires were highly inter-correlated, and we found no statistically reliable differences in the strength or patterns of correlations between flexibility (i.e., cognitive, and affective task-switching ability) and the three different measures of self-rated resilience. Even when not significant, all correlations were negative, suggesting greater flexibility (more efficient task switching) in more resilient persons. Even though it cannot be excluded that with higher statistical power subtle differences between correlation strengths might be detectable, the lack of such differential effects is consistent with the strong inter-correlations among the three questionnaire measures. These results, thus, suggest that psychological flexibility may be associated to a strong shared component of resilience independent of the specific conceptualization of resilience. In line with this reasoning, the overall correlation pattern did not change when combining individual questionnaires into a composite score (see Supplementary S1). However, given the high correlation of the three resilience measures, the question arises to what extent different aspects of resilience are at all validly and distinguishably measurable by means of self-report questionnaires. In the present study, questionnaires were administered in a sample of healthy young individuals, which were not specifically selected for having experienced stressful or traumatic events. Particularly in case of the BRS it might thus be questionable whether the self-rated ‘perceived ability’ to recover from negative life events matches the ‘real life’ adaptation to adversity when actually confronted with highly negative experiences or whether it does not also reflect aspects of the personality. In addition, a study by Waaktaar & Torgersen (2010) found that the Resilience scale by Wagnild & Young (1993) did not outperform a Big Five personality inventory in predicting adaptive behaviors (e.g., global life satisfaction, quality of relationships) in adolescents. Furthermore, positive affect (Haglund et al. 2007) and positive appraisal style (Kalisch et al. 2015) have been shown to be important factors for resilience. Thus, it is possible that self-report resilience questionnaires do not capture resilience-specific characteristics beyond favorable personality traits or more general resilience-conducing factors such as positive affect, especially in individuals who have not experienced severe life stress. Therefore, it would be important for future research to examine the extent to which resilience measures other than self-report questionnaires are related to flexibility. Recent approaches are for example, to measure resilience in terms of an outcome by assessing the level of psychosocial functioning in response to actual stress experiences (e.g., using recently introduced regression-based approaches; cf. Booth et al. 2020; Kalisch et al. 2021; see also Elman et al. 2022, for methodological considerations) or by analyzing the individual trajectory of psychological recovery in the aftermath of traumatic distress (Bonanno et al. 2011; Galatzer-Levy, 2018).

### Theoretical Implications

The present work aimed at understanding in more depth the frequently postulated association between resilience and psychological flexibility, whereby flexibility was variably associated with diverse demands including the flexible regulation of emotions or efficient problem solving (Haglund et al. 2007; Kashdan 2010; Parsons et al. 2016). A flexible person may be better equipped with cognitive resources to handle stressful events. More precisely, cognitive flexibility or flexible cognitive control processes allow to actively guide attentional processes, e.g., towards positive aspects of events, and to choose appropriate appraisal and or coping mechanisms that will eventually result in a more effective processing of the aversive situation and consequently in higher resilience (Parsons et al. 2016). This reasoning receives support from empirical observations in psychiatric conditions, in which affected individuals tend to show inflexible – rigid – behavior patterns (e.g., rumination in depression; Nolen-Hoeksema et al. 2008). Whereas rigidity is considered an extreme endpoint on a ‘flexibility continuum’ (Kashdan 2010) our correlative results from a sample of unaffected young adults point in the same direction, i.e., that behavioral flexibility vs. inflexibility is associated with greater vs. lower resilience (and thus, by conjecture, mental health). At the same time, the absence of an association with distractor inhibition (cognitive stability) indicates that resilience is not associated to better executive functioning in general, but that it is specifically related to psychological flexibility or the flexible use of cognitive control processes. This finding is in line with previous results showing that working memory, another aspect of executive functioning, is unrelated to trait resilience (Genet & Siemer 2011).

For understanding the relationship between psychological flexibility and mental health, we consider it one of the most fundamental challenges to clarify whether resilience is specifically associated to flexibly handling affective experiences, potentially even specific to affectively negative experiences, or whether resilience is more generally related to flexible cognitive processing. A study focusing on anxiety and worry came to the conclusion that anxiety may not be related to general impairments in cognitive flexibility, but rather to specific difficulties when shifting from non-affective to affective aspects of positive stimuli and to greater flexibility when shifting attention away from affective aspects of negative information (Twivy et al. 2020). Our results for affective switch costs speak against a specific association of resilience with disengaging from negative information. However, our regression results highlight affective flexibility as opposed to cognitive flexibility as critical for resilience to stress and adversity. For this association, emotion regulation flexibility could be an important link. Emotion regulation flexibility has repeatedly been shown to be associated with good mental health (Aldao et al. 2015). While an association between affective flexibility and emotion regulation has a high face validity, there exists little research that directly investigates this relationship. Two studies in healthy subjects reported that affective flexibility was related to the use of emotion regulation strategies in experimental settings (Grol & De Raedt 2021; Johnson 2009b). Furthermore, a study with euthymic patients with bipolar disorder showed that emotion regulation capacity measured by questionnaire predicted response time switch costs in an affective flexibility paradigm (Gul & Khan 2014).

Also other constructs related to emotion regulation (like rumination or reappraisal effectiveness) have been shown to be associated with affective flexibility (Genet et al. 2013; Malooly et al. 2013). Future research is needed to explore in more depth the relationship between affective flexibility, emotion regulation flexibility, and other cognitive processes critical for resilience.

### Limitations and future direction

In the present work, we provide robust evidence for theoretically proposed associations between resilience and affective flexibility. In doing so, we have replicated previous results and extended previous work by including multiple resilience questionnaire that highlight different aspects of resilience. However, it is not clear to what extent self-report questionnaires can really capture the different concepts of resilience in a differentiated way. Furthermore, for statistical comparisons of the correlations between flexibility indices and the three resilience measures, the study may have been underpowered to detect potentially subtle differences and thus cannot provide any definite conclusions. Future work should, therefore, explicitly integrate also alternative conceptions of resilience, for example by using longitudinal data which considers the outcome of adversity or traumatic experiences (Bonanno et al. 2015).

In the rather limited number of experimental studies on affective flexibility that have so far been reported, there exists a relative heterogeneity of specific design choices in the experimental procedures, including different stimulus materials or consistent or inconsistent response button mapping (see above). As a consequence, results have been quite variable with respect to some correlative associations (e.g., with non-affective task switching costs as well as with respect to the dependency of effects on specific valence conditions). We accordingly suggest that it is of great importance to study the effects of such differences in experimental paradigms more systematically. Also, while asymmetric affective switch costs have now been reported repeatedly (Eckart et al. 2021; Reeck & Egner 2015), the exact mechanisms underlying this phenomenon are not fully understood and future work should for example aim at clarifying the complementary roles of negative priming effects vs. endogenous cognitive control.

Last, we also think that for a refined understanding of the relationship between cognitive or affective flexibility and resilience, it may be beneficial to study in more depth the relationship between flexibility and other cognitive processes critical for resilience, in particular emotion regulation.

## Conclusion

Our results suggest a relationship between cognitive as well as affective task switching efficiency and psychological resilience, which was, however, more pronounced for affective than non-affective (i.e., cognitive) flexibility. Future research is needed to understand the precise mechanisms behind this relationship, eventually with the long-term goal of developing intervention options to promote resilience.

## Supplementary Information

Below is the link to the electronic supplementary material.

Supplementary file1 (DOCX 1002 KB)

## Data Availability

All data and script files are available on the project page https://osf.io/v6y39/. The preregistration can be found at https://osf.io/u47yg

## Footnotes

1 Note that the analysis of switch costs was not the primary focus of the present study. For a more detailed statistical analysis, as well as a comparison with an additional flexibility task, we refer the reader to Kraft et al. (2020). Note also that response times and error rates vary minimally between these two analyses (compare Table 2 of this report with Table 1 of Kraft et al., 2020), due to differences in the criteria for performance-based subject exclusion.

2 Three persons made no correct response when switching from a happy face in the gender task to an angry face in the emotion task, one person when switching from an angry face in the gender task to an angry face in the emotion task.
