# Supplementary material for: Individual differences in resilience to stress are associated with affective flexibility
Source: Psychol Res. 2022 Dec 17;87(6):1862–79. doi: 10.1007/s00426-022-01779-4 (PMC10366320; doi:10.1007/s00426-022-01779-4)
Supplement: Supplementary file 1 — Supplementary file1 (DOCX 1002 KB) [file 426_2022_1779_MOESM1_ESM.docx]

**Supplemental material**

**S1 Correlations of response time-based measures of flexibility/stability with a composite score of the three resilience measures**

|  |  |
| --- | --- |
| *Affective flexibility task (N=99)* |  |
| Switch costs emotion→gender | ***r_s_*=-.22, *p*=.01** |
| Switch costs gender→emotion | ***r_s_*=-.31, *p*<.001** |
| *Cognitive flexibility task* |  |
| Switch costs (N=94) | ***r_s_*=-.20, *p*=.03** |
| Distractor inhibition costs (N=91) | *r****_s_***=.07, *p*=.24 |
| Spontaneous switching (N=83) | *r****_s_***=.01, *p*=.46 |
|  |  |

All tests are one-sided. The composite score was calculated by z-standardizing and summing up data of the three resilience questionnaires used in the study.

**S2 Supplemental figure. *Correlations of resilience scores and response time switch costs split according to the valence of the preceding stimulus and the valence of the stimulus in the switch trial.***

**
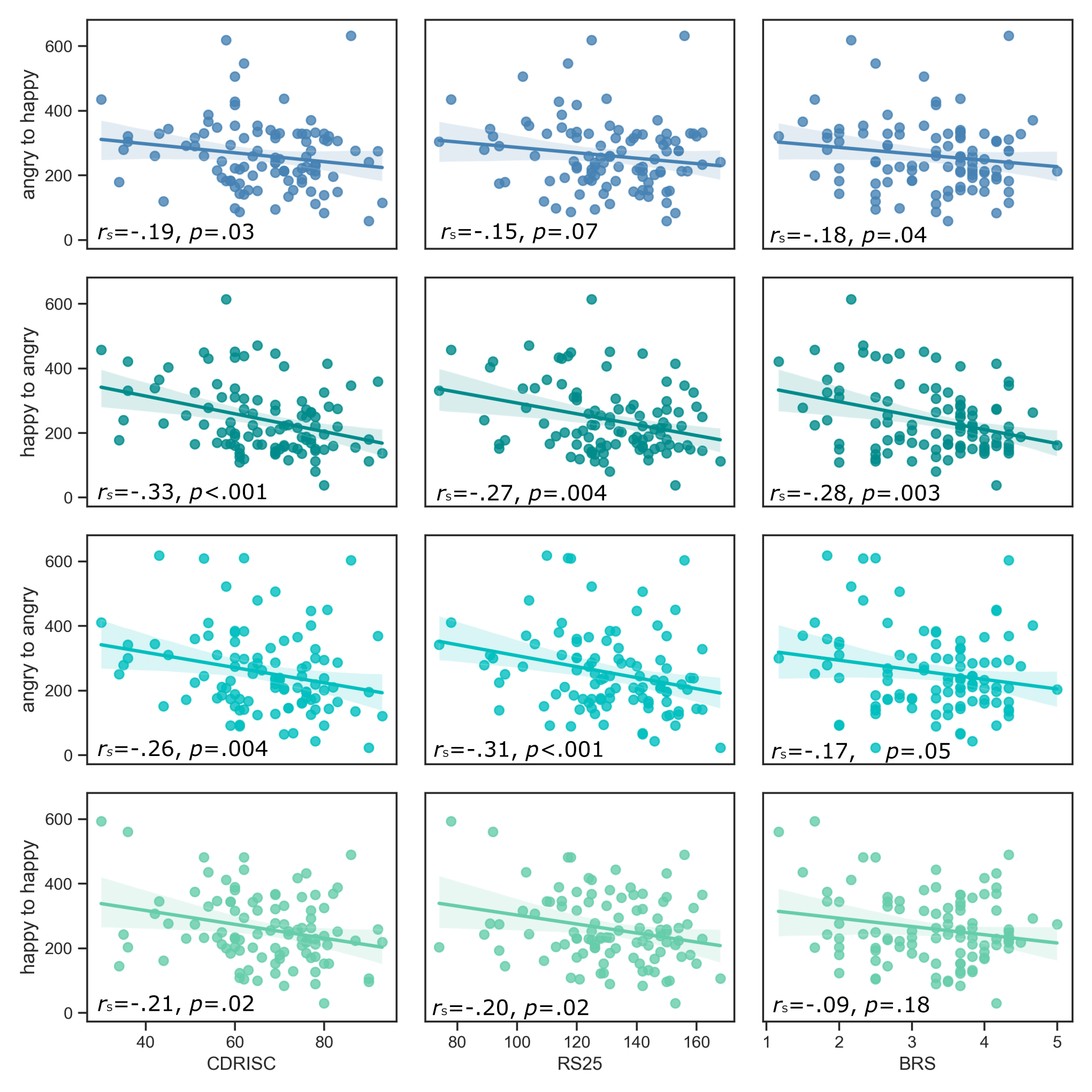
**

***S3 Analyses with covariates***

According to our pre-registered analysis plan, gender, age, handedness, drug use, and history of psychiatric disorder were considered as possible confounding variables. Switch costs in both paradigms were affected by gender of the participants: Female subjects showed higher switch costs (cognitive: 381 ms; affective: gender → emotion: 294 ms, emotion → gender: 273 ms) compared to male subjects (cognitive: 307 ms, t (92) = 2.84, *p =* .006; affective: gender → emotion: 245 ms, t (97) = 2.05, *p* = .04; emotion → gender: 219 ms, t (97) = 2.88, *p* = .005). Regarding error rate switch costs, men showed higher costs (11.2%) during switches from the gender to the emotion task than women (5.3%, t (97) = 2.56, *p* = .01), but no other gender effects were found. Furthermore, cognitive switch costs differed between right-handed (N=88, 352 ms) and left-handed participants (N=6, 217 ms, t (93) = 2.80, *p* = .006), but due to the substantial inequality of cell sizes, this may be a spurious result. No further influences were found from gender, age, handedness, drug use, or history of psychiatric disorder on affective or cognitive switch costs, or on distractor inhibition costs or the spontaneous switch rate in the cognitive flexibility paradigm. Resilience scores were found to be higher in subjects without history of psychiatric disorder (N=80; CD-RISC: 68.1; RS-25: 133.0; BRS: 3.44) compared to participants with lifetime history of a psychiatric disorder (N=10; CD-RISC: 57.2, t (88) = 2.52, *p* = 0.01; RS-25: 113.7, t (88) = 3.11, *p* = .002; BRS: 2.42, t (88) = 3.99, *p* < .001; note that 10 persons provided no data on previous psychiatric disorders). Gender, age, handedness, or drug use were not correlated with resilience scores.

Because of gender effects on switch costs and effects of psychiatric history on resilience scores, the correlation analyses of switch costs and resilience scores (Hypotheses 1 and 1a) were rerun as partial correlations correcting for gender and psychiatric history, but results did not change (Supplemental Table S3.1). Also, the inclusion of handedness did not change correlations between cognitive switch costs and resilience scores (Supplemental Table S3.2).

**Supplemental table S3.1. *Partial correlations of response time-based measures of flexibility and resilience questionnaires controlling for gender and psychiatric history.***

|  | CD-RISC | RS-25 | BRS |
| --- | --- | --- | --- |
| Affective flexibility (N=99) |  |  |  |
| Switch costs emotion→gender | **r_s_=-.20, p=.02** | **r_s_=-.18, p=.036** | r**_s_**=-.10, p=.17 |
| Switch costs gender→emotion | **r_s_=-.30, p=.001** | **r_s_=-.28, p=.003** | **r_s_=-.21 p=.02** |
| *Cognitive flexibility* |  |  |  |
| Switch costs (N=94) | **r_s_=-.22, p=.02** | r_s_=-.15, p=.08 | r**_s_**=-.14, p=.09 |

All tests are one-sided. Statistically significant results (based on critical p-values determined using Dubey-Armitage-Parmar correction for three endpoints; see Methods section for details) are printed in bold. CD-RISC = Connor-Davidson Resilience Scale; RS-25 = Resilience Scale; BRS = Brief Resilience Scale.

**Supplemental table S3.2. *Partial correlation of response time-based cognitive flexibility and resilience questionnaires controlling for gender, psychiatric history, and handedness of the participants.***

|  | CD-RISC | RS-25 | BRS |
| --- | --- | --- | --- |
| *Cognitive flexibility* |  |  |  |
| Switch costs (N=94) | **r_s_=-.21, p=.02** | r_s_=-.16, p=.06 | r**_s_**=-.12, p=.13 |

All tests are one-sided. Statistically significant results (based on critical p-values determined using Dubey-Armitage-Parmar correction for three endpoints; see Methods section for details) are printed in bold. CD-RISC = Connor-Davidson Resilience Scale; RS-25 = Resilience Scale; BRS = Brief Resilience Scale.
